# Pattern of recurrence and survival after D2 right colectomy for cancer: is there place for a routine more extended lymphadenectomy?

**DOI:** 10.1007/s13304-022-01317-2

**Published:** 2022-07-01

**Authors:** Matteo Palmeri, Andrea Peri, Valentina Pucci, Niccolò Furbetta, Virginia Gallo, Gregorio Di Franco, Anna Pagani, Chiara Dauccia, Camilla Farè, Desirée Gianardi, Simone Guadagni, Matteo Bianchini, Annalisa Comandatore, Gianluca Masi, Chiara Cremolini, Beatrice Borelli, Luca Emanuele Pollina, Giulio Di Candio, Andrea Pietrabissa, Luca Morelli

**Affiliations:** 1 General Surgery Unit, Department of Translational Research and New Technologies in Medicine and Surgery, University of Pisa, Italy, Via Paradisa 2, 56125 Pisa, Italy; 2 Department of General Surgery, University of Pavia, 27100 Pavia, Italy; 3 Medical Oncology Unit, Fondazione IRCCS Policlinico San Matteo, 27100 Pavia, Italy; 4 Oncology Unit, Department of Translational Research and New Technology in Medicine and Surgery, University of Pisa, 56124 Pisa, Italy; 5 Division of Surgical Pathology, University-Hospital of Pisa, 56124 Pisa, Italy; 6 EndoCAS (Center for Computer Assisted Surgery), University of Pisa, 56124 Pisa, Italy

**Keywords:** Colon cancer, Right colectomy, D2 standard lymphadenectomy, Complete mesocolic excision, D3 lymphadenectomy, Surgery

## Abstract

**Background:**

Conventional Right Colectomy with D2 lymphadenectomy (RC-D2) currently represent the most common surgical treatment of right-sided colon cancer (RCC). However, whether it should be still considered a standard of care, or replaced by a routine more extended D3 lymphadenectomy remains unclear. In the present study, we aim to critically review the patterns of relapse and the survival outcomes obtained from our 11-year experience of RC-D2.

**Methods:**

Clinical data of 489 patients who underwent RC-D2 for RCC at two centres, from January 2009 to January 2020, were retrospectively reviewed. Patients with synchronous distant metastases and/or widespread nodal involvement at diagnosis were excluded. Post-operative clinical–pathological characteristics and survival outcomes were evaluated including the pattern of disease relapse.

**Results:**

We enrolled a total of 400 patients with information follow-up. Postoperative morbidity was 14%. The median follow-up was 62 months. Cancer recurrence was observed in 55 patients (13.8%). Among them, 40 patients (72.7%) developed systemic metastases, and lymph-node involvement was found in 7 cases (12.8%). None developed isolated central lymph-node metastasis (CLM), in the D3 site. The estimated 3- and 5-year relapse-free survival were 86.1% and 84.4%, respectively. The estimated 3- and 5-year cancer-specific OS were 94.5% and 92.2%, respectively.

**Conclusions:**

The absence of isolated CLM, as well as the cancer-specific OS reported in our series, support the routine use of RC-D2 for RCC. However, D3 lymphadenectomy may be recommended in selected patients, such as those with pre-operatively known CLM, or with lymph-node metastases close to the origin of the ileocolic vessels.

## Introduction

The presence of metastatic lymph-nodes is one of the most important prognostic factors among patients with radically resected right sided colon cancer (RCC) and is a major driver for the administration of adjuvant chemotherapy [1–3]. In recent decades, principles of surgical oncological radicality have changed significantly together with a greater understanding of the prognostic factors and various new techniques have been introduced, ranging from the ‘no-touch technique’ to the D3 lymphadenectomy obtained during the so-called Complete Mesocolic Excision (CME-D3). First described by Hohnberger in 2009, CME-D3 technique appears to offer the potential for harvesting a greater number of lymph nodes (≥ 28) by performing a more extensive lymphadenectomy [4–6]. Hence, the claim is made that by allowing the removal of more metastatic lymph-nodes, it could achieve better prognostic outcomes when compared with conventional surgery, therefore, challenging the routine use of the conventional Right Colectomy with D2 lymphadenectomy (RC-D2) [7].

Another main goal of CME-D3 is to gain more appropriate oncological dissection that includes embryological fascial planes, by translating the concept of Total Mesorectal Excision (TME) from rectal surgery [8]. However, while the principles of TME had found as main driver the well-known role of local recurrence as “the oncological issue” after surgery for rectal cancer, facing the RCC, the background does not seem so clearly comparable in this regard [9–11].

In recent years, many studies have compared different techniques (open, laparoscopic, or robotic) RC-D2 versus CME-D3, and some authors advocate the latter as possible gold standard in RCC [12–22]. To date, however, CME-D3 has not been widely accepted as a standard of care, also due to the technically challenging nature of the procedure, which is associated with higher intra-operative complications and postoperative morbidity [23].

This controversy, aimed us to critically review the oncological results obtained from our 11-year two-centre experience of RC-D2 for non-metastatic RCC, with a particular view to the pattern of recurrence and the survival curves, particularly to obtain information whether, in a long-term oncologic follow-up, were evidence of recurrences theoretically avoidable if a CME-D3 would have been performed as standard technique in all patients.

## Materials and methods

### Patient selection

A retrospective analysis was conducted involving patients who had undergone a RC-D2 for non-metastatic cancer at two surgical centres, in Pisa and Pavia (Italy), from January 2009 to January 2020. The population for this study was obtained from the electronic institutional prospectively maintained databases, and data were retrospectively analysed.

Patients with histologically confirmed RCC who underwent open, laparoscopic, or robotic surgery were included. Patients with synchronous distant metastases and/or widespread nodal involvement at diagnosis, beyond the origin of the ileocolic vessels, and those without follow-up were excluded. Patients who had undergone to emergency procedures, and those who had developed relapse within 1 month after surgery were also excluded. The study was approved by the institutional review boards.

### Data collection

Pre-operative evaluation included demographic information (age, gender), body mass index (BMI), American Society of Anaesthesiologists (ASA) score, and the value of tumour markers Ca 19–9 and CEA. Surgical data included timing of intervention (elective or emergency), type of surgical approach (laparoscopic, robot-assisted, or open), rate of conversion to open surgery for minimally invasive cases, and surgical time. Pathological data referred to size, pathological stage (p ‘tumour, node, metastases [TNM]) and differentiation grade of tumours, the number of harvested lymph-nodes, and the number of positive lymph-nodes detected.

The analysed postoperative data were length of hospital stay (LOS), postoperative complications (based on Clavien–Dindo classification), and oncological outcomes evaluated at follow-up [24].

### Surgical technique

A conventional right colectomy using a laparoscopic or robot-assisted (‘medial to lateral’), or laparotomic (‘lateral to medial’) approach with D2 lymphadenectomy is performed.

First, the lower margin of the ileocolic vessels is identified and a ≈ 2 cm window of the mesentery is opened. We extend laterally to reach the lower margin of the ascending mesocolon which is then medially opened to reach up to the root of the ileocolic vessels located at the right margin of the superior mesenteric vein (SMV). The ileocolic vessels and the right colic vein and right colonic artery are isolated and divided at their root close to the SMV, and the surrounding lympho-adipose tissue is dissected. Proceeding in a cephalad direction towards the radix of the middle colonic vessels, the middle colonic artery and vein are transected at their root and nearby lymph-nodes are dissected, based on the location of the tumour (right flexure and transverse colon). The extent of resection includes the terminal ileum, caecum, ascending colon, hepatic flexure, and proximal transverse colon with proximal and distal margins ≥ 5 cm from the lesion.

### Adjuvant therapy

Adjuvant chemotherapy was administered based on the pathological stage of the disease and patients’ general condition and comorbidities, based on medical oncologists’ choice as per their clinical practice.

Adjuvant chemotherapy was not recommended in stage I resected CRC patients.

The combination of fluoropyrimidine (5-fluorouracil or capecitabine) plus oxaliplatin was the preferred choice in stage III and high-risk stage II resected CRC patients. High-risk stage II was defined by the presence of at least one risk factors (perforation, occlusion, T4, lympho-vascular invasion, G3–4 and < 12 nodes examined).

In stage II patients without risk factors, adjuvant chemotherapy with fluoropyrimidine alone could be proposed.

In patients with high-risk stage II and III unfit for combination, fluoropyrimidine alone might be an alternative therapeutic option, while exclusive follow-up was reserved to those with contraindication to chemotherapy administration.

### Follow-up

After surgery, patients were followed-up for post-surgical evaluation at 7 days and at 1 month after discharge. The oncologic follow-up visits were scheduled every 6 months for the first 5 years following surgery, and included blood examination, abdomen US or total body CT scan (once per year), colonoscopy.

When analysing the follow-up data, recurrences were classified in nodal only, extra-nodal only and nodal plus extra-nodal. Furthermore, particular attention was paid to the pattern of nodal recurrence, by distinguishing between widespread lymph-node metastases (WLM) and central lymph-node metastases (CLM), i.e., those in the territory of the D3 lymphadenectomy described by CME-D3.

Relapse-free survival (RFS) was defined as the time between surgical resection and disease recurrence or the last follow-up in case of alive patients with no evidence of disease; deaths without recurrence were censored at the time of death. Recurrence was defined as radiological evidence of intra-abdominal, enhanced, soft tissue around the surgical site or of distant metastases, including lymph-node relapse. Overall survival (OS) was defined as the time from colon surgery to death due to any cause, or to last follow-up for alive patients. Patients who died within 30 days of undergoing surgery were not included in the survival analysis. Cancer-specific OS (csOS) was defined as the time between surgery and death in relapsed patients, or last follow-up. Patients died without recurrence where censored at the time of death.

### Statistics

The SPSS^®^ Statistics (v.24) software program was used to conduct statistical analyses. Continuous data with normal distribution are expressed as mean ± standard deviation. The Kaplan–Meier method was applied to plot survival curves and to estimate DFS and OS rates; *p* < 0.05 was considered a statistically significant result.

## Results

### Patient characteristics

A total of 489 non metastatic patients underwent a RC-D2 during the study period. Among them, 400 patients were enrolled in the study, as 81 patients were excluded, because lost at the follow-up, developed relapse or died for surgical or medical complications within 1 month after surgery. Further eight patients were excluded, because had undergone to emergency procedures.

In hospital mortality of the entire series, including emergency procedures, was 1% (5/489 patients), and was, respectively, due to: an anastomotic leakage, a post-operative bleeding in a cirrhotic patient, a myocardial infarction, a pneumonia and a pulmonary embolism.

Among the 400 patients finally enrolled in the study, 191 (47.8%) were male, the mean BMI was 25.2 ± 3.9 kg/m^2^ and mean age was 71.8 ± 11.5 years. Regarding ASA score, 22 patients (5.5%) were ASA 1, 216 patients (54%) ASA 2, 146 patients (36.5%) ASA 3 and 16 patients (4%) ASA 4.

### Surgical outcomes

The mean surgical time was 198.9 ± 64.1 min. In 344 (86%) patients, no post-operative complications occurred. Postoperative morbidity was 14%. According to Clavien–Dindo score we registered 19 (4.7%) grade I, 24 (6%) grade II, 3 (0.7%) grade IIIa and 8 (2%) grade IIIb, 2 (0.5%) grade IVa. Complications are summarized in Table 1.

**Table 1:** Post-operative complications

No Complications, *n* (%)	344 (86%)
Anemia which necessitated a blood transfusion, *n* (%)	14 (3.3%)
Postoperative ileus, *n* (%)	10 (2.8%)
Anastomotic leak requiring re-operation, *n* (%)	8 (2%)
Wound infection, *n* (%)	4 (1%)
Abdominal abscess requiring percutaneous drainage, *n* (%)	3 (0.8%)
Intestinal obstruction, *n* (%)	2 (0.5%)
Evisceration requiring surgical intervention, *n* (%)	1 (0.2%)
Undetermined fever, *n* (%)	4 (1%)
Cardiovascular complications, *n* (%)	5 (1.2%)
Pulmonary complications, *n* (%)	5 (1.2%)

In 252 patients (63%) we performed a minimally invasive approach (227 with pure laparoscopy and 25 with robot-assistance) with a conversion rate to open surgery of 1.5%. The ileo-colic anastomosis was hand-sewn in 284 cases (71%) or stapled in 116 cases (29%). The median time of hospital stay was 8 [6–11] days.

### Pathological characteristics

The tumor site was: caecum in 170 cases (42.5%), ascending colon in 167 patients (41.7%), hepatic flexure in 38 patients (9.5%) and proximal transverse colon in 25 cases (6.3%). The tumour size was > 4 cm in 230 patients (57.5%). The mean number of total lymph nodes harvested was 25.9 ± 12.7. The mean number of metastatic lymph nodes harvested was 1.4 ± 3.5.

The grading was G1 in 3 patients (0.7%), G2 in 267 cases (66.7%) and G3 in 130 patients (32.6%).

Concerning the pathologic T parameter, 2 lesions (0.5%) were classified pTis, 27 (6.7%) pT1, 69 (17.3%) pT2, 262 (65.5%) pT3, and 40 (10%) pT4. Regarding the pathologic N parameter, 261 (65.2%) were classified pN0, 81 (20.2%) pN1, and 58 (14.6%) pN2. Globally, our cohort included 261 (65.2%) stages I–II, and 139 (34.8%) stage III patients.

### Oncological outcomes

One hundred and fifty-one patients (37.8%) underwent adjuvant therapy, being oxaliplatin-based doublets the most frequent choice. In particular, among them, 68 patients (45%) received the combination of fluoropyrimidine plus oxaliplatin, 64 patients (42.4%) fluoropyrimidine alone and for 19 patients (12.5%) the adjuvant schedule was unknown.

At a median follow-up of 62.0 months (range 57–139 months), 91 (22.7%) patients died and 55 (13.8%) experienced disease relapse.

**Fig. 1:**
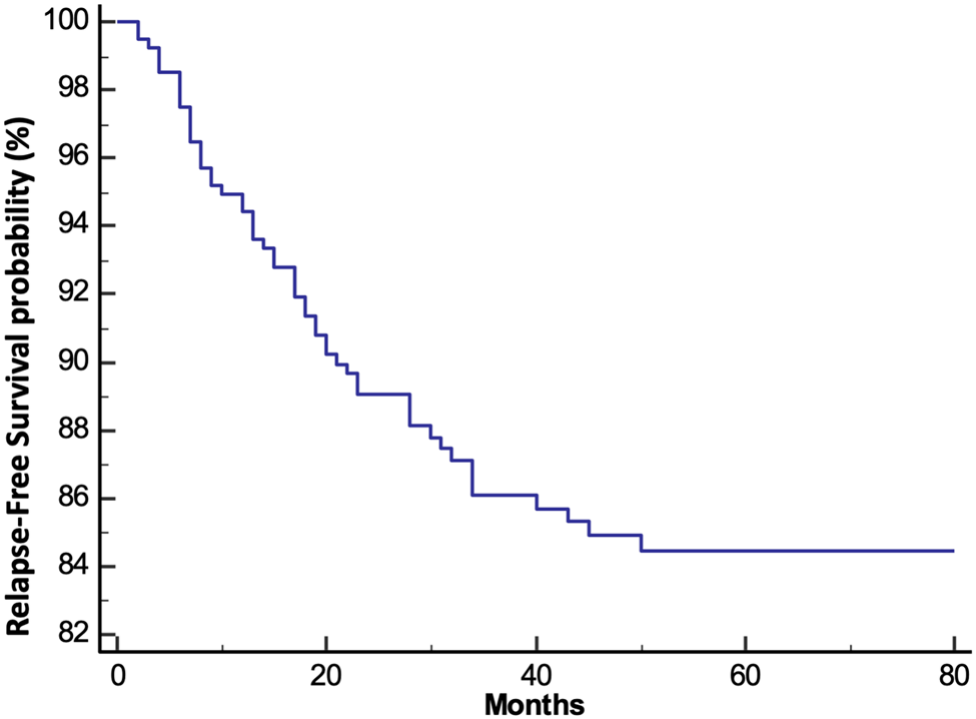
Relapse free survival (RFS) determined by Kaplan–Meier curves

**Fig. 2:**
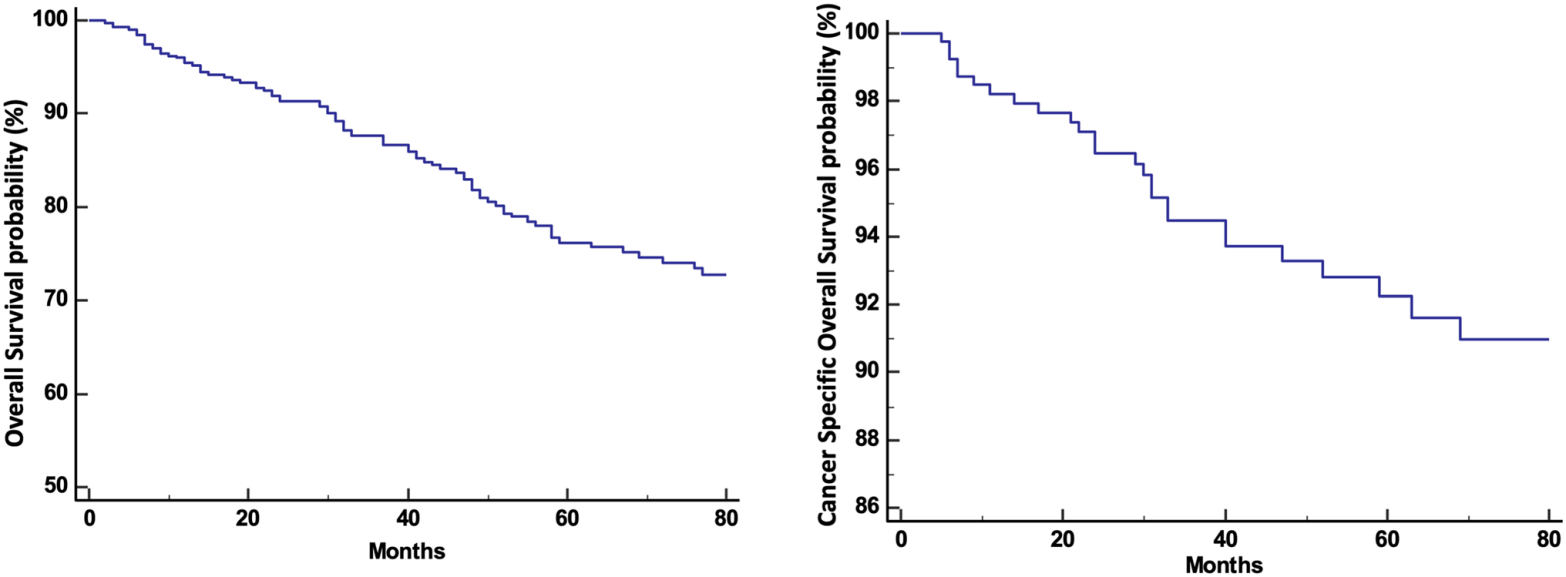
Overall survival (OS) and cancer-specific overall survival (cOS) determined by Kaplan–Meier curves

**Fig. 3:**
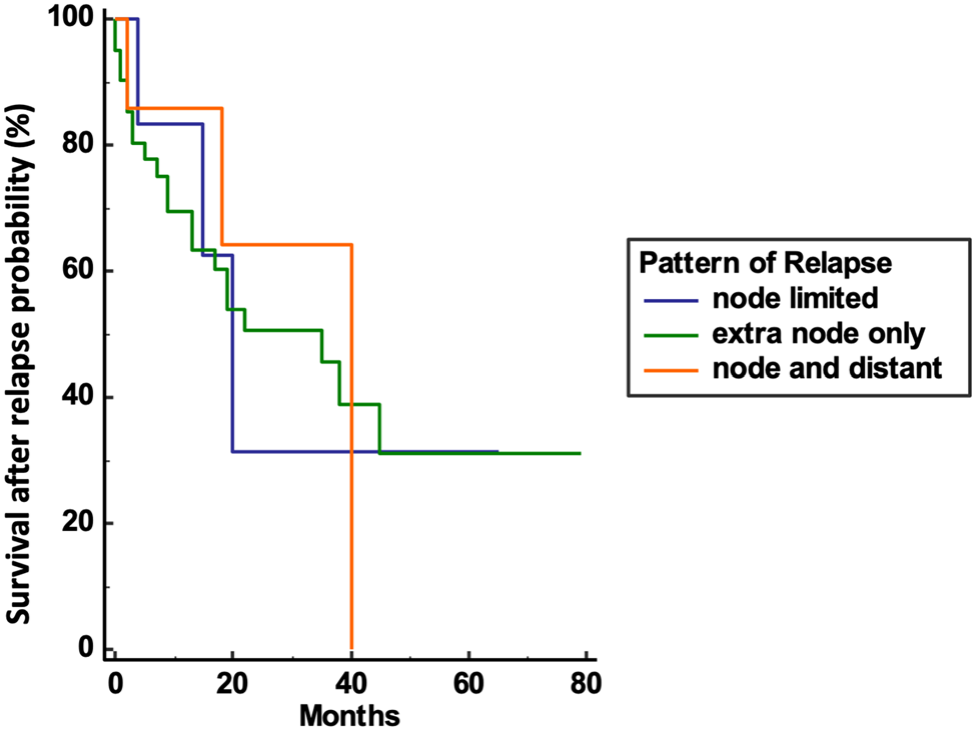
Survival after relapse determined by Kaplan–Meier curves according to the pattern of recurrence

Two patients (3.6%) out of 55 patients had recurrence at the ileocolic anastomosis, 40 (72.7%) presented distant extra-nodal metastases (lung, liver, bone, and peritoneum), 7 (12.8%) had both extra-nodal and nodal metastases, and 6 (10.9%) had only WLM (para-aortic, intercaval aortic, mesenterial, and iliac vessels); none had isolated CLM. Three- and five-year RFS rates were 86.1% and 84.4%, respectively (Fig. 1). Three- and five-year OS rates were 87.6% and 76.2%, while 3- and 5-year csOS were 94.5% and 92.2%, respectively (Fig. 2). No differences in term of survival after relapse were reported according to the different patterns of relapse considered (nodal only, extra nodal or both) (*p* = 0.94) (Fig. 3). Dividing patients per stage, 3- and 5-year RFS rate were 92.1% and 90.8% in stages I–II and 74.5% and 72.3% in stage III, respectively. Three- and five-year OS rate were 90.9% and 80.3% in stages I–II and 81.3% and 68.6% in stage III, respectively, while 3- and 5-year csOS were 97.5% and 96.7% in stages I–II and 88.7% and 83.5% in stage III, respectively.

## Discussion

The presence of metastatic lymph-nodes is an independent factor for determining the prognosis of RCC. A higher number of harvested lymph-nodes during surgery is claimed to increase surgical radicality, allowing for proper disease staging, and, accordingly, gaining better oncological outcomes and overall patients survival [1, 2, 25–29].

In this regard, although the degree of evidence is weak, by considering the minimum recommended harvested lymph-nodes reported in oncologic literature [25, 30–33], D2 lymphadenectomy performed during a right colectomy, through ligation at the origin of the ileo-colic vessels and by removing all the tissue on the right-side of the SMV, is historically considered appropriate [34, 35].

Nevertheless, as in other surgical fields (e.g., gastric and pancreatic surgery), due to improvements in surgical techniques, over time, authors have proposed a modified local dissection that included embryological fascial planes, by translating the concept of TME from rectal surgery [8], with a more extended lymphadenectomy, by describing the CME-D3 technique, with the expectation of obtaining a better prognosis, also for RCC patients [36–41].

The increasing number of authors supporting CME-D3, and the rising trend over the past number of years among some groups proposing CME-D3 as a new possible standard of care, prompted us to conduct a critical review of our clinical and oncological results with RC-D2, in a long-term follow-up.

In our experience, we successfully performed CME-D3 in selected cases, e.g., in patients with CLM at diagnosis, or in particularly young patients with ileo-colic node involvement; however, as we did not reach a sufficient number to make a meaningful comparison between the two groups, we, therefore, excluded these patients and instead focused on the D2 lymphadenectomy group. Accordingly, in the present study, we analysed short and long-term outcomes for patients who had undergone RC-D2 in a large series over 11 years with a long follow-up, and we focused on disease recurrence and its influence on OS through the assessment of csOS. In particular, we aimed to detect if a theoretically more extensive dissection, such as that proposed using CME-D3, could confer the possibility of a better oncological prognosis among patients who had experienced a cancer recurrence.

Interestingly, while CLM rates in the literature have been reported in a range from 0 to 5.8% [42], and the rates of true skip metastases to D3 nodes (i.e., metastases in the D3 area without metastases in the D1 area, close to the tumour) range from 0.8 to 2% [43], in our series, CLM was not reported. Indeed, all the registered recurrences referred to systemic metastases, to WLM, or both, but without cases involving CME-D3 site.

In terms of survival, the results reported in the literature remain conflicting. In fact, several studies reported that harvesting a greater number of lymph-nodes is not necessarily related to longer survival [44, 45]. Furthermore, despite the theoretical advantages of CME-D3, the meta-analyses by Alhassan et al. [46] and Wang et al. [36] were both unable to definitively conclude whether CME-D3 conferred a statistically significant improvement in long-term oncological outcomes compared with conventional lymphadenectomies. Some other articles indicated instead an improvement in OS when using CME-D3 (100% at 5 years [28]; 93.5% at 3 years [29]; 81.6% at 3 years [1]); conversely, other authors [45] did not observe a significant increase in survival (2–3.1%) for stages I, II, and III in the case of CME-D3, and reported a 5-year OS of 56.1%. On the other hand, recent studies [47], including a meta-analysis by De Simone et al. [21], and a systematic review by Mazzarella et al. [22], showed an improvement of CME-D3 in long-term oncological impact reporting up to 85% 5-year OS, but included studies reporting oncological results defined by the same authors as “unbelievable” because of the 100% in OS at any stage of cancer [28] or no recurrence cases in stage I, II and III [48, 49].The latter study could be also biased in favour of the CME-D3 group by the heterogeneity of the samples (50% of CME-D3 patients were at stage II and only 20% at stage III, whereas the 35% of the non-CME-D3 patients were at stage III).

Local and distant recurrence following CME-D3, attested in these reviews, is 12.25%, without a significant difference in 3-year and 5-year DFS, between CME-D3 and non-CME-D3. Analysing in deep these results, it is still unclear if the improvement in cancer-related survival in CME-D3 can be explained solely by a more aggressive loco-regional surgery [39]. Indeed, also the evolution in adjuvant chemotherapy as well as the growing surgical experience and patient centralization in high-volume centres may have contributed to improve colon cancer prognosis and have played as confounding factors [21, 22, 50]. Moreover, another important bias can be related to the frequent missing report in literature of the type of recurrence (nodal, distant, or both) and most importantly, the pattern of nodal recurrence (WLM or CLM), increasing complexity of data interpretation. Another bias of these studies is the heterogenous numerosity of the samples evaluated from the authors, with only few articles reporting on a large study population, and several restricted case series, which could have influenced the favourable outcomes.

In our study of 405 patients, 13.8% of them experienced local and distance recurrence, in line with published retrospective series, and most importantly, none of patients, with a median follow-up of 63 months, had experienced a CLM, although 34.6% had a significant risk of relapse, being stage III patients. Moreover, as no differences in term of survival after relapse were reported according to the different patterns of relapse, it is unlikely that a more extensive dissection, such as that proposed using CME-D3, could have conferred a better oncological prognosis.

OS and RFS indicated similar results to those reported in the literature focused on CME-D3, since we registered 3-year OS of 87.0% and 5-year OS of 74.8%, while the 3- and 5-year RFS were 85.9% and 84.3%, respectively. Moreover, results concerning 3- and 5-year csOS are quite reassuring (94.3% and 92%, respectively), since OS in retrospective analysis involving patients treated during a decade, could be biased by non-related tumor deaths.

In particular, and in line with the described pattern of recurrence, comparing 3-year survival of our series to the OS of CME-D3 reported by the most recent study [1, 21, 22] on the subject, no significant improvement was confirmed.

A further critical consideration is related to the risk/benefit ratio of routinely performing the CME-D3 in RCC surgical treatment, to carefully balance the potential complications related to this technique with the prognostic advantages in terms of survival [23, 51]. In the literature, the comparison between CME-D3 and conventional surgery underscores additional critical aspects [52]. One of these is represented by the increased surgery times and a steep learning curve for performing CME-D3, resulting in increased surgical and anaesthesiologic stress for the patient. Moreover, this issue could lead to a shift away from surgery that is widespread in peripheral hospitals and within the performance capabilities of younger surgeons toward surgery that can only be performed by highly experienced surgeons within referral centres [53]. Authors [28] have also reported a median surgery time of 239.7 min in the case of CME-D3, while our data indicate 196 min for performing RC-D2. Furthermore, the CME-D3 technique also appears to be associated with increased rates of intraoperative complications; in particular, a high risk of vascular injury has been reported with an incidence of 1.6% [54, 55]. This type of intra-operative complication, in line with the reported data of standard right colectomy in the literature, was not observed in our series.

Bertelsen et al. [23] noted that the rate of injury to other organs observed during resection was significantly more common in the case of the CME-D3 technique (9.1% in a CME-D3 group versus 3.6% in a non-CME-D3 group). Again, in our study, these types of complications were not observed. Finally, a further undesired consequence of a more central dissection may be an increased incidence of chylous fistula, as the literature reports a rate of 2.5% in CME-D3 procedures [56].

In our opinion, the reduced and not significant incidence of CLM, such as skip lesions, balanced with the higher incidence of major intraoperative complications as reported in the literature [54], as well as our long-term follow-up results, should be considered not in favour of a routine CME-D3, at least in most cases of I–II and III-stage colon cancer lesions, for which conventional surgery with D2 lymphadenectomy seems safe and appropriate.

Obviously, as our study did not evaluate the outcomes of young non-metastatic patients with known positive lymph-nodes close to the origin of the ileocolic vessels, or those with pathological nodes next to superior mesenteric vessels known at surgery, as they were treated with CME-D3, our observations should not be extended to these cases.

The main limitations of this study are its retrospective nature, the long temporal window, and its dual-centre design with different learning curves for surgeons, particularly when introducing minimally invasive approaches. Another limitation is a lack of a control group with CME-D3 in our series to enable directly comparing the two techniques.

Currently, two prospective randomized studies, RELARC and COLD, are ongoing and particularly oncologic results are expected to give a more decisive contribution to this pending and controversial issue [54, 57].

## Conclusions

The absence of isolated CLM, as well as the csOS reported in our series, is in favour of the routine use of RC-D2 for RCC. However, a CME-D3 may be the treatment of choice if carried out by expert surgeons and among a smaller cluster of patients, such as young individuals with pre operatively known lymph-node metastases close to the origin of the ileocolic vessels or CLM.
